# Unusual Tumors Causing Extrahepatic Portal Venous Obstruction

**DOI:** 10.1155/1996/72709

**Published:** 1996

**Authors:** B. C. Sharma, V. A. Saraswat, R. K. Dhiman, U. C. Ghoshal, A. S. Puri, S. S. Sikora

**Affiliations:** 1 Department of Gastroenterology Sanjay Gandhi Post Graduate Institute of Medical Sciences Lucknow India; 2 Department of Surgical Gastroenterology Sanjay Gandhi Post Graduate Institute of Medical Sciences Lucknow India

## Abstract

Extrahepatic portal vein obstruction has been reported to be associated with tumors of liver, bile ducts and pancreas. We report two cases, one with gastric leiomyosarcoma and another with Non Hodgkin’s lymphoma, complicated by portal vein block and presenting with gastric variceal bleeding. Portal vein block in both cases was due to direct vascular infiltration. Development of portal hypertension posed difficulties in management.


*HPB Surgery*, 1996, Vol.9, pp.165–167
Reprints available directly from the publisher
Photocopying permitted by license only

© 1996 OPA (Overseas Publishers Association)
Amsterdam B.V. Published in The Netherlands
by Harwood Academic Publishers GmbH
Printed in Malaysia

### Unusual Tumors Causing Extrahepatic Portal Venous Obstruction

B.C. SHARMA, V.A. SARASWAT, R.K. DHIMAN, U.C. GHOSHAL, A.S. PURI and S.S. SIKORA*

Departments of Gastroenterology and *Surgical Gastroenterology, Sanjay Gandhi Post Graduate Institute of Medical Sciences, Lucknow

*(Received 26 March 1994)*

Extrahepatic portal vein obstruction has been reported to be associated with tumors of liver, bile ducts and pancreas. We report two cases, one with gastric leiomyosarcoma and another with Non Hodgkin's lymphoma, complicated by portal vein block and presenting with gastric variceal bleeding. Portal vein block in both cases was due to direct vascular infiltration. Development of portal hypertension posed difficulties in management.

KEY WORDS: Extrahepatic portal venous obstruction   Non-Hodgkin's lymphoma   gastric leiomyosarcoma   portal vein thrombosis   portal vein

### INTRODUCTION

Neoplastic diseases are known to cause splenic and portal venous thrombosis. Tumors reported to present in this manner usually arise from the liver or pancreas.[1,2] We report two patients presenting with recurrent major bleeds from gastric varices secondary to splenic and portal vein thrombosis developing as a complication of gastric leiomyosarcoma in one patient and Non-Hodgkin's lymphoma in the other.

### CASE-1

A 50 years old man presented with a history of upper abdominal pain and recurrent episodes of melena, along with anorexia and weight loss for three months. There was no history of vomiting, hematemesis, jaundice or ascites. Physical examination revealed severe anemia, moderate hepatosplenomegaly and an epigastric mass extending to the left hypochondrium. Laboratory data showed hemoglobin 4 g/dl, total leucocyte count 4400/ul, ESR 78 mm, total bilirubin 8.5 μmol/l, AST 40 IU/L, ALT 26 IU/L, alkaline phosphatase 600 IU/L (normal 70-110), serum proteins 6 g/dl with albumin of 3 g/dl. Upper gastrointestinal endoscopy revealed a polypoid mass just below fundus on the posterior wall of stomach with large tumor like gastric varices. The mucosa showed severe portal hypertensive gastropathy in the body of the stomach. Endosonography confirmed the presence of both gastric varices and a large mass arising from posterior wall below the fundus of stomach CT Scan of abdomen revealed an exophytic growth arising from fundus and body of stomach and also involving pancreas, splenic vein, spleen and retroperitoneum. Histopathological examination of endoscopic biopsies from the tumor revealed round cells with bizarre, pleomorphic, hyperchromatic nuclei, prominent eosinophilic nucleoli and moderate cytoplasm extending in thin processes at places. Areas of hemorrhage and necrosis were present and twenty to thirty mitotic figures per ten high power fields could be seen. On the basis of these finding, the diagnosis of gastric leiomyosarcoma causing splenic vein thrombosis and with extensive retroperitoneal infiltration was made. As the growth was unresectable and no surgical option for palliation was available, the patient was treated with blood transfusion and supportive measures only and was discharged from hospital at his request.

*Correspondence to* Dr. V.A. Saraswat, Department of Gastroenterology, Sanjay Gandhi Postgraduate Institute of Medical Sciences, Raebareli Road, P.B No. 375 Lucknow 226 014. India

### CASE II

A 60 years old woman presented with history of fever, hematemesis, melena and splenomegaly for one month accompanied by significant anorexia and weight loss. There was no history of jaundice, encephalopathy or dependent edema. Physical examination revealed severe anemia, generalized cervical and axillary lymphadenopathy, moderate hepatosplenomegaly and ascites. There were no stigmata of chronic liver disease. Laboratory data showed : hemoglobin 6 g/dl, total leucocyte count 17000/ul P70% and L30%, total bilirubin 17 µmol/l, AST 40 IU/L, ALT 18 IU/L and alkaline phosphatase 85 IU/L. Ultrasound scan of abdomen showed multiple enlarged preaortic and paraaortic lymphnodes with another huge lymph node mass seen in the region of the tail of pancreas. The splenoportal axis could not be visualized. Upper gastrointestinal endoscopy revealed grade II oesophageal varices with blood oozing from large fundal varices. Axillary node biopsy confirmed diagnosis of Non-Hodgkin's lymphoma, diffuse and predominantly small cleaved cell type. Ascitic fluid was deep straw coloured with protein 3.9 gm/dl, sugar 1.04 g/l total leucocyte count 52/ul, with 90% lymphocytes. Exfoliative cytology of ascitic fluid also showed plentiful lymphoid cells. In view of persistent and recurrent variceal bleeding the patient was subjected to gastric devascularization. Operative findings showed splenomegaly with a 15×10 cm lymph node mass in the region of body and tail of pancreas, compressing the portal vein and infiltrating the splenic flexure of the colon. The short gastric vessels were dilated. The liver was grossly enlarged firm and smooth with no evidence of cirrhosis or lymphomatous infiltration on peroperative wedge biopsy. Bleeding was controlled after surgery . However the patient continued to have ascites, deepening jaundice and she died of liver failure 6 weeks after surgery.

### DISCUSSION

The etiology of extrahepatic portal vein obstruction (EHPVO) is unknown in most patients. But may be secondary to increase in blood coagulability, inflammatory changes in the vessels or associated neoplasms in older patients.[3] In Western series neoplasms have been found to account for 13% to 24% of patients with portal vein block.[1,4] Pancreatic cancer is the commonest such malignancy and accounts for about 11% of cases whereas about 5% are due to hepatocellular carcinoma.[1,4] In some series hepatocellular carcinoma is reported to be the most frequent cause of malignant portal vein obstruction.[2] Other tumors associated with portal vein block are cholangiocarcinoma, retroperitoneal and colonic lymphoma, primary hepatic lymphoma, enlarged metastatic lymph nodes and gastric adenocarcinoma.[1–12]

Portal vein obstruction in patients with neoplasms can be due to direct invasion of portal vein by tumor, extrinsic compression of portal vein, portal vein thrombosis due to tumor thrombus or hypercoaguable state associated with malignancy[7,10]. Apart from causing EHPVO, portal hypertension in lymphoma may be due to liver involvement or increased blood flow secondary to splenomegaly.[13–14] In the first case, gastric leiomyosarcoma produced splenoportal thrombosis by direct invasion of splenic vein, whereas in the second case, EHPVO could have been due to both external compression of portal vein and infiltration of splenoportal venous system by the lymphoma.

The clinical picture in such cases is dominated by features of the primary disease. In a recently published series from Japan, 75% of cases had initial symptoms and signs attributable to primary disease.[15] Unlike our cases, none of the patients actually presented with complications of portal hypertension. These authors suggested that the sonographic appearance of the portal vein is different in malignant EHPVO when compared to those having EHPVO secondary to benign disease or idiopathic EHPVO. In EHPVO of malignant etiology, the portal trunk is clearly identified with apparent occlusion such as membranous obstruction, extrinsic compression or thrombus like obliteration with surrounding cavernomatous structures, whereas in patients with idiopathic EHPVO or those secondary to benign disease, the portal trunk is replaced by a cavernomatous, hyperechoic band with blurred demarcation.[15]

Araki *et al.*[10] described four cases of portal vein block associated with gastric adenocarcinoma. In these cases portal vein block was due to portovenous tumor thrombi detected by imaging techniques. In the present case of gastric leiomyosarcoma splenic vein thrombosis was probably due to direct invasion and no tumor thrombi could be detected in the splenic vein on CT Scan. The exophytic pattern of growth of the leiomyosarcoma explains direct invasion of the splenic vein, which was not seen in the four cases of gastric adenocarcinoma[10] because these were mainly intraluminal growths.

Extrahepatic portal venous obstruction due to such tumors results in portal hypertension which can complicate surgery by causing profuse bleeding from portovenous collaterals. Jerome *et al.*[16] reported three cases of cholangiocarcinoma with portal vein obstruction, where bleeding from collaterals led to added complications during surgery. They suggest preoperative evaluation of the portal system, if there is clinical or endoscopic evidence of portal hypertension. Endoscopically gastric varices may look like tumor as seen in the present case of leiomyosarcoma. There can be difficulty in differentiating such gastric varices from tumors in the fundus of stomach and endosonography is useful in this situation before biopsying such lesions[17]. In the present case of leiomyosarcoma tumor biopsy was taken after confirming solid tumor by endosonography.

In both patients reported here, splenoportal axis thrombosis resulted in large fundal varices with recurrent episodes of major variceal bleeding. Control of variceal bleeding in these patients posed a difficult surgical challenge, as seen in the first case where, due to extensive tumor spread, no surgical treatment was possible. Where technically feasible, gastric devascularization with splenectomy would be the procedure of choice. However, even in the second case, a large lymphnode mass at the splenic hilum made splenectomy difficult and the procedure had to be confined to gastric devascularization.

### REFERENCES

1. Macpherson A.I.S. (1984) Portal hypertension due to extrahepatic portal vein obstruction. A review of forty cases. *J Royal CoLL Surg Edinb*, **29**: 4 -10.
2. Suguira N, Ohto M, Kimura K, Okuda K, Kondou F and Hirooka N. (1986) Diagnosis and clinical features of portal vein tumor thrombosis in hepatocellular carcinoma. *Jpn J Gastroenterol*, **83**: 2151–2160.
3. Sahni P, Pande G.K, Nundy S. (1990) Extrahepatic portal vein obstruction. *Br J Surg*, **70**: 1201–1202.
4. Witte C.L, Brewer M.L, Witte M.H *et al.* (1985) Protean manifestations-of Pylethrombosis–A review of thirty four patients. *Ann Surg*, **202**: 191–202.
5. Albacete R.A, Mathews M.J, Saini N. (1967) Portal vein thrombosis in malignant hepatoma. *Ann Intern Med*, **67**: 337–348.
6. Legmann P, Ollier P, Barge J *et al.* (1985) Primary lymphoma of liver: an exceptionally rare tumor. Ultrasonography and computed tomography imaging in one case and reveiw of literature. *J Radiol*, **66**: 599–602.
7. Cohen J, Edelman R, Chopra S. (1992)Portal Vein thrombosis. *Am J Med*, **92**: 173–182.
8. Penek J, Webber B.L, Kirsch R.E. (1976) Portal vein thrombosis with ascites. A case report. *Afr Med J*, **50**: 173–176.
9. Subramanyam BR, Balthazar EJ, Lefieur RS *et al.* (1984) Portal thrombosis corelative analysis of sonography, CT and angiography. *Am J Gastroenterol*, **79**: 773–776.
10. Araki T, Suda K, Sekikawa T *et al.* (1990) Portal venous tumor thrombosis associated with gastric adenocarcinoma. *Radiology*, **174**: 811–814.
11. Seenu V, Goel A.K, Shukla N.K *et al.* (1994) Hodgkin's lymphoma of colon: An unusual cause of isolated splenic vein obstruction. *Indian J Gastroenterol*, **13**: 76–78.
12. Madsen M.S, Petersen T.H, Sommer H. (1986) Segmental portal hypertension. *Ann Surg*, **204**: 72–77.
13. Shaldon S, Sherlock S. (1962) Portal hypertension in the myeloproliferative syndrome and the reticuloses. *Am J Med*, **32**: 758–764.
14. Lindor K, Rakela J, Perrault J, Van Heerden J. (1987) Non cirrhotic portal hypertension due to lymphoma- Reversal following splenectomy. *Dig Dis Sci*, **32**: 1056–1058.
15. Sugiura N, Shouichi M, Masao O *et al.* (1993) Extrahepatic portal vein obstruction in adults detected by ultrasound with frequent lack of portal hypertension signs. *J Gastroenterol Hepatol*, **8**: 161–167.
16. Jerome A, Sastman H.D, Morton I *et al.* (1979) Cholangiocarcinoma with portal vein obstruction. *Radiology*, **130**: 15 -20.
17. Ruttenberg D, Graham S, Burns D *et al.* (1991) Abdominal tuberculosis- A cause of portal vein thrombosis and portal hypertension. *Dig Dis Sci*, **36**: 112–115.

